# Measuring Antibiotic Stewardship Programmes and Initiatives: An Umbrella Review in Primary Care Medicine and a Systematic Review of Dentistry

**DOI:** 10.3390/antibiotics9090607

**Published:** 2020-09-16

**Authors:** Leanne Teoh, Alastair J Sloan, Michael J McCullough, Wendy Thompson

**Affiliations:** 1 Melbourne Dental School, University of Melbourne, Carlton, Victoria 3053, Australia; alastair.sloan@unimelb.edu.au (A.J.S.); m.mccullough@unimelb.edu.au (M.J.M.); 2 Division of Dentistry, University of Manchester, Manchester M13 9PL, UK; wendy.thompson15@nhs.net

**Keywords:** antimicrobial stewardship, antimicrobial resistance, primary care, medical, dental, outcome assessment, healthcare, antibiotics

## Abstract

Antibiotic stewardship aims to tackle the global problem of drug-resistant infections by promoting the responsible use of antibiotics. Most antibiotics are prescribed in primary care and widespread overprescribing has been reported, including 80% in dentistry. This review aimed to identify outcomes measured in studies evaluating antibiotic stewardship across primary healthcare. An umbrella review was undertaken across medicine and a systematic review in dentistry. Systematic searches of Ovid Medline, Ovid Embase and Web of Science were undertaken. Two authors independently selected and quality assessed the included studies (using Critical Appraisal Skills Programme for the umbrella review and Quality Assessment Tool for Studies with Diverse Designs for the systematic review). Metrics used to evaluate antibiotic stewardship programmes and interventions were extracted and categorized. Comparisons between medical and dental settings were made. Searches identified 2355 medical and 2704 dental studies. After screening and quality assessment, ten and five studies, respectively, were included. Three outcomes were identified across both medical and dental studies: All focused on antibiotic usage. Four more outcomes were found only in medical studies: these measured patient outcomes, such as adverse effects. To evaluate antibiotic stewardship programmes and interventions across primary healthcare settings, measures of antibiotic use and patient outcomes are recommended.

## 1. Introduction

Antimicrobial resistance is a serious global public health problem responsible for increased healthcare costs and poorer clinical outcomes [1]. The World Health Organisation (WHO) global action plan on antimicrobial resistance aims “to ensure, for as long as possible, continuity of successful treatment and prevention of infectious diseases with effective and safe medicines that are quality-assured, used in a responsible way, and accessible to all who need them” [2]. All Member States have been urged to develop nation action plans aligned with the objectives of the global action plan [3] and a governance framework of these plans is being produced, including arrangements for monitoring and evaluation [4].

While antimicrobials include antibiotic, antiviral and antifungal medicines, most antibiotic prescribing occurs in primary healthcare [5,6], with dentistry accounting for an estimated 10% of international usage [7,8]. Unnecessary prescribing of antibiotics is widespread with studies in both United States (US) and United Kingdom (UK) showing 80% use not in accordance with guidance [9,10,11,12].

Antibiotic stewardship programmes (ASPs) in primary care are essential to try to curb antibacterial resistance [13]. Antibiotic prescribing behaviour is known to be context specific and ASPs appropriate for use in secondary care services (hospital inpatient and outpatient settings) may be less relevant for primary healthcare services delivered by generalists in community settings [14]. Similarly, evaluation of ASPs and interventions must be careful to ensure they measure both the intended benefits as well as potential unintended negative consequences [15]. Whilst outcome measures of antibiotic usage have been published by international multidisciplinary groups, the authors were aware of a more extensive set of metrics used in some studies of primary healthcare [6,16].

This review aimed to identify a suite of outcome measures used in published studies to evaluate ASPs and interventions across primary care medicine and dentistry. An umbrella review (systematic review of systematic reviews) was used to identify outcome measures from the numerous studies across primary care medicine and a systematic review was used to determine the metrics from dentistry, where fewer studies have been published [14]. Comparison of the outcomes measured between medical and dental settings enabled opportunities to inform the evaluation of new antibiotic stewardship interventions by researchers, and support evaluation of ASPs by healthcare providers in primary medical and dental care.

## 2. Method

### 2.1. Protocol and Research Questions

The protocol for this two-part systematic review was registered in PROSPERO [17]. Both reviews conformed to the Preferred Reporting Items for Systematic Reviews and Meta-Analyses (PRISMA) statement. The first research question “What is known from published systematic reviews about outcomes employed to measure outcomes in antibiotic stewardship programmes/interventions across primary medical care?” was addressed by an umbrella review. The second question: “What is known from the published literature about metrics employed to measure outcomes in antibiotic stewardship programmes/interventions for dental care?” was addressed by undertaking a systematic review. Primary care was defined as “the first point of contact in the healthcare system, including general practice medicine and dental services” [14,18].

### 2.2. Search Strategies and Study Selection

In May 2020, three databases were searched from their earliest dates: Ovid Embase, Ovid Medline and Web of Science. The search strategies and terms were developed in consultation with an information specialist at the University of Melbourne. The search terms and strategy for both reviews are shown in Supplementary Material Tables S1 and S2. The search strategies used a “human” search limit as animal studies were not eligible for the reviews and an “English language” limit due to a lack of resources for translation.

Research studies published in peer-reviewed journals were included if they reported measurement of ASP or interventions to optimise antibiotic use in non-specialist primary medical or dental care. Studies related to specialist care delivered in primary care settings (e.g., hospital outpatients and ambulatory care) and/or primary care services delivered in hospital settings were excluded. Studies which did not include antibiotic prescribing were also excluded.

**Figure 1:**
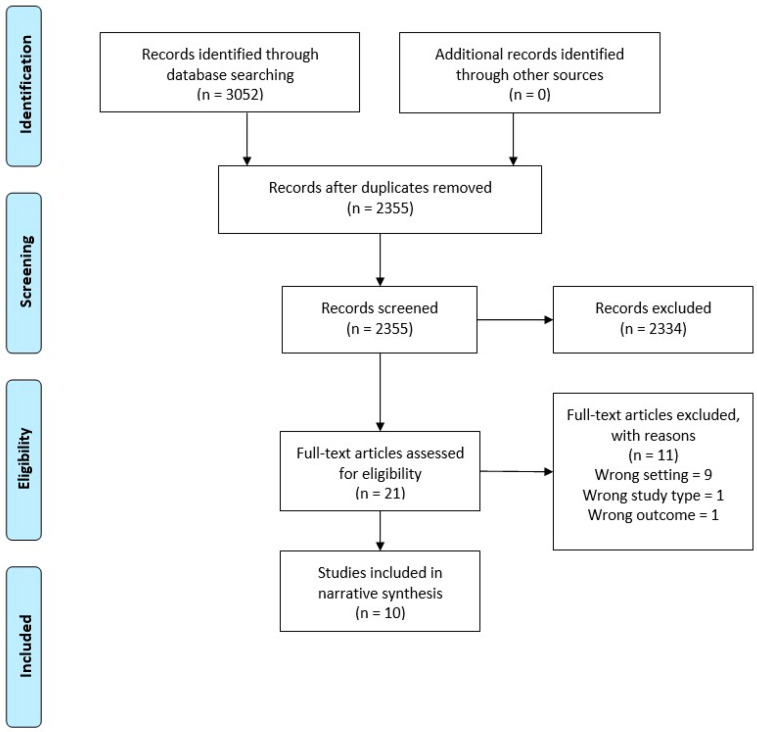
Preferred Reporting Items for Systematic Reviews and Meta-Analyses (PRISMA) Flow Diagram—Umbrella Review.

**Figure 2:**
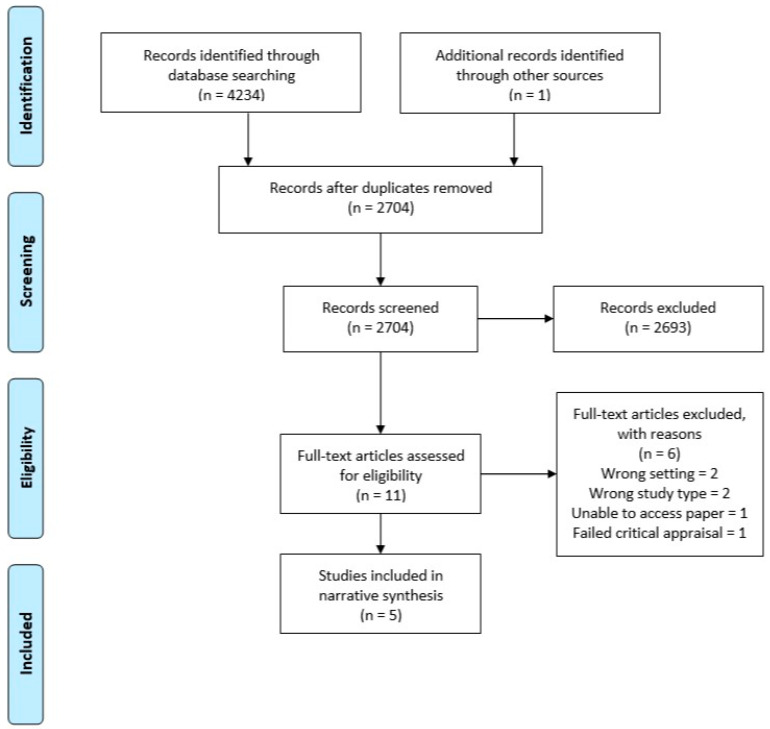
PRISMA flow diagram—systematic dental.

After performing the search using each database, the titles and abstracts were extracted into Endnote X9 and duplicates were removed. Separate Endnote libraries were created for the umbrella review and the systematic review. Two authors (LT and WT) screened all titles and abstracts independently for potential inclusion. Full text of all the shortlisted studies were assessed independently for eligibility by LT and WT. Discrepancies were resolved through discussion. Summaries of the selection processes used for the umbrella and systematic reviews are shown in Figure 1 and Figure 2, respectively.

### 2.3. Quality Assessment

The Critical Appraisal Skills Programme (CASP) Checklist for Systematic Reviews was used assessing the quality of studies included in the umbrella review (Supplementary Material Table S3) [19]. The validated 16-item Quality Assessment Tool for studies with Diverse Design (QATSDD) for the systematic review for quality assessment of included studies. (Supplementary Material Table S4) [20]. These tools were conducted separately by both LT and WT and discrepancies were resolved through discussion. Studies that scored less than 50% in the QATSSD assessment failed the quality assessment and were not included in the systematic review.

### 2.4. Extraction of Outcome Measures

Measures used to evaluate ASPs and interventions in both the umbrella review in primary medical care and the systematic review of primary dental care were extracted by two authors (LT and WT) independently. Disagreements were resolved through discussion. Categories were developed by one author (LT) to group the outcome measures and checked independently with another author (WT). Outcomes that did not relate to the aims of this review were excluded from the extraction process.

### 2.5. Comparing the Outcome Measures

After completion of extracting and categorising the measures used to quantify the ASP outcomes, these metrics were compared and contrasted across both settings (primary medical care vs dental care).

## 3. Results

### 3.1. Study Selection

For the umbrella review across primary medical care, 3052 studies were identified for possible inclusion in the review. After duplicates were removed, 2355 were screened, 21 eligible for full text review, and eleven of these excluded with reasons. This resulted in ten articles that met the inclusion criteria and were of adequate quality (Figure 1).

For the systematic review across primary dental care, 4234 studies were identified for possible inclusion in the review. After duplicates were removed, 2704 were screened, 11 eligible for full text review, and six of these excluded with reasons. This resulted in five studies that met the inclusion criteria and were of adequate quality (Figure 2).

### 3.2. Study Characteristics

#### 3.2.1. Umbrella Review across Primary Medical Care

The characteristics of the included studies in the umbrella review are shown in Table 1 [21,22,23,24,25,26,27,28,29,30]. The included systematic reviews encompassed 109 individual primary research studies with a range of publication dates (1992–2016). Participants were general practitioners, and adults, children and parents of child patients with common infections such as respiratory tract and urinary tract infections. The ASPs and interventions being evaluated included point-of-care testing, the use of real-time epidemiology and pharmacy-led interventions. Analysis of the overlap between these 109 studies found 13 appeared in two or more of the systematic reviews (Supplementary Material Table S5).

#### 3.2.2. Systematic Review across Primary Dental Care

The characteristics of the five studies included in the systematic review across primary dental care are shown in Table 2 [31,32,33,34,35]. The study participants were all dentists: four of the studies specified general dental practitioners and one did not specify the type of dentists included. The author of the latter study was contacted to confirm that only general dentists participated in the study [35]. The studies were published from 2006–2020. The ASPs and interventions being evaluated included: audit and feedback, education, advice from the health board, dissemination of guidelines and implementation of a dental-specific prescribing tool.

#### 3.2.3. Outcomes Measured in Primary Medical Care

Eight ASPs and intervention outcomes were identified (as shown in Table 3) with a total of 27 individual outcomes measured in primary medical care. Details of the individual factors from of the systematic reviews included in the umbrella review across primary medical care are presented in Table 1. Hu et al., 2016 [25] included 13 studies in their systematic review and 12 in their meta-analysis. Only the results for the latter 12 were presented, so these were included in this review.

#### 3.2.4. Outcomes Measured in Primary Dental Care

Four outcomes measured to evaluate ASPs and interventions were identified, as shown in Table 3. Details of the individual factors from each of the primary research studies included in the systematic review of primary dental care are presented in Table 2. Whilst Seager et al., 2006 [34] attempted to use patient satisfaction as an outcome measure, data collection was discontinued due to a low rate of questionnaire return.

#### 3.2.5. Comparing the Outcomes Measured between Primary Care Medicine and Dentistry

Three outcome measures were found across both primary medical and dental care studies. These all measured antibiotic usage: by quantity (number), rate and quality (including appropriateness).

Four outcome measures were unique to primary medical care and related to patient outcomes and experiences: re-consultation rates, adverse effects, severity of symptoms, and patient reported outcomes such as satisfaction. One outcome measure was unique to dentistry: The confidence and attitude of dentists towards the intervention (Table 3).

## 4. Discussion

An extensive list of outcomes measured in studies of primary medical and dental care to evaluate ASPs and interventions has been collated. It presents options for researchers testing new interventions in primary medical and dental care as well those wishing to evaluate the implementation of ASPs across primary healthcare. It should also enable the translation of existing metrics to other primary care settings, including from medicine to dentistry. While most ASPs have focused on various healthcare settings (such as hospital, ambulatory and outpatient settings) [6,16,36,37], this is the first comprehensive study to focus on measuring the outcome of ASPs and interventions in non-specialist primary medical and dental care.

### 4.1. Antibiotic Stewardship and Resistance

Whilst antibiotic stewardship was introduced as a way to tackle antibiotic resistance [38], this review has shown that most studies, especially in dentistry, focus primarily on reducing antibiotic usage. The reason is that it is impractical to measure directly a change in the resistance profile of bacteria associated with an antibiotic stewardship intervention and to relate cause (reduce antibiotic prescribing) and effect (reduced antibiotic resistance) [39]. Correlating the timing of the antibiotic exposure to the development of resistance further compounds the difficulty of measuring resistance. Furthermore, the development and selection of antibiotic resistance is influenced by a range of factors, including antibiotic use in agriculture, aquaculture as well as in healthcare. In addition, socio-economic factors may affect rates of resistance, such as overcrowding of urban areas, poor infection control in healthcare facilities and the availability of substandard quality of antibiotics [1,40].

By contrast antibiotic usage is relatively easy to measure and various international studies have demonstrated that a reduction in antibiotic use can result in changed patterns of resistance to antibiotics. A Cochrane systematic review has demonstrated that the prescribing of an antibiotic in primary care for a respiratory or urinary infection results in patients developing bacterial resistance to that antibiotic that may persist for up to 12 months [41]. At the population level, decreased consumption of macrolide antibiotics in Finland resulted in decreased levels of resistance of group a streptococci to erythromycin [42]. It is understandable, therefore, why all of the studies included in these reviews employed measures of antibiotic usage as a proxy measure for tackling antibiotic resistance rather than measuring it directly.

### 4.2. Antibiotic Usage versus Quality of Antibiotic Use

Antibiotic usage as expressed as quantity, rates, percentage and relative ratios was commonly used across both medical and dental primary care. As these outcome measures are easy to interpret and monitor, they are used widely across healthcare settings [39]. A systematic review followed by a multidisciplinary consensus procedure determined a range of metrics to measure quantity of antibiotic use in outpatient settings [16]. These included the defined daily dose (DDD)/1000 patients/day metric, which is often used for international comparison of antibiotic consumption [43] as defined by the World Health Organisation [44], and was also used in the randomised trial employed across all NHS dental practices in Scotland to measure antibiotic use [32]. However, quantity measurements based on the DDD unit is based on standard dosing and regimen to a 70kg adult [16]. It is therefore not suitable for measuring antibiotic use in children and only provides a rough estimate of use and comparisons between countries [16,45]. The difficulty arises as guidelines and practice differ between places and over time. Furthermore, whilst DDD might be useful for researchers and for international comparisons, for practical use in primary care it is less relevant. The number of antibiotic courses started is potentially more relevant than DDD as it indicates the number of patients exposed to antibiotics rather than the total amount of antibiotics used [46].

### 4.3. Clinical and Patient Outcome Measures

A key finding of this study was that none of the dental studies employed clinical or patient outcome measures, such as adverse effects to evaluate ASPs. A study of antimicrobial stewardship in outpatient settings drew similar conclusions, that most of the metrics related to antibiotic usage rather than patient or clinical outcomes [6]. Incorporating patient-related or clinical outcomes alongside measures of antibiotic use brings the metrics identified closest to the definition of antimicrobial stewardship employed in Australia—to reduce harm whilst also curtailing the incidence of antibiotic resistance [47].

Infections of the head and neck may rapidly become life-threatening if they spread into and along the oropharyngeal fascia, such as the pharyngeal space [48]. With the increasing incidence of antibiotic resistant infections, infections which are treated by antibiotics alone (rather than with a surgical intervention to remove the source of the infection) will continue to spread unabated. It is anticipated that there will be an increased in the incidence of systemic conditions such as sepsis and the resurgence of Lemierre’s syndrome (a rare but potentially severe condition involving suppurative thrombophlebitis of the internal jugular vein) is anticipated [49,50].

With rates of antibiotic resistance differing between places and over time, and as there is wide variety in the way clinical services are provided in different parts of the world, including between high and low-middle income countries, selecting the right outcome measures for the context is vital. One set of metrics will not suit all primary healthcare settings and so it is recommended that this suite of clinical and patient outcome metrics be used or adapted when evaluating future ASPs and interventions. Whilst many of the clinical and non-clinical factors that influence antibiotic prescribing by clinicians in primary medical and dental care (including patient expectations and workload), there are also differences between the contexts [14]. Further research is indicated to test which of the clinical and patient outcome metrics identified from medical studies will best measure outcomes in the dental context whilst guarding against negative unintended consequences.

Measures of quantity are crucial as they provide baseline measures for monitoring and tracking antibiotic use, but they do not provide insight into the quality of patient care. Reduced antibiotic usage does not always correlate with improved clinical outcomes and the significance of changes in antibiotic prescription rates is not well understood [26]. For this reason, the Infectious Diseases Society of America (IDSM) has recommended using outcome measures that focus concurrently on the quality (appropriate indication and regimen, such as antibiotic type, dose, duration, route) as well as quantity of antibiotic prescribing to ensure that individual patients have been treated appropriately [51]. Similar to other areas of clinical dentistry where patient-centred outcomes to measure various aspects of oral health are recommended [52], metrics that incorporate both antibiotic use as well as clinical outcomes are preferable.

Interestingly whilst all of the studies in this review measured quantity of antibiotic use, only four of the systematic reviews across medical care [24,26,29,30] and three studies in dental care [31,33,35] reported using the metric “in accordance with guidelines”. The appropriateness of the guidelines will therefore affect the quality of prescribing. Guidelines also change over time: The recent Australian dental antibiotic guidelines have recommended an extended spectrum of antibiotics coverage for acute odontogenic infections that is in contrast with the remainder of the international dental community [53]. Guidelines also vary between countries; the antibiotic prophylaxis guidelines for infective endocarditis in the UK [54] is different compared to Australia [55] and the US [56]. It is therefore recommended that indicators of both quality and quantity should be used to evaluate ASPs and interventions. More than one quantity should be considered to gain a better understanding of antibiotic use [16].

Incorporating clinical outcomes in addition to antibiotic use and quality antibiotic use is important to provide further insight to the effectiveness of the ASPs and interventions on individual patient care. For example, a recently published dental intervention pre–post study showed a decrease in antibiotic use and an overall decrease in inappropriate prescribing, but a significant number of prescriptions were still for inappropriate indications [35]. Clinicians have poor adherence to guidelines as socio-behavioural theory has been shown that they tend to form internalised, tacit guidelines, continuously modified by personal experience, leaders in the field and continuing education [57]. Several factors influence antibiotic prescription and are different for each context [14]. Due to high rates of unnecessary antibiotic prescribing, it is expected that ASPs and interventions that target inappropriate prescribing would naturally see a concurrent reduction in antibiotic use. Furthermore, since it is accepted that the use of broad-spectrum antibiotics contributes more towards antibiotic resistance due to the increased selection of bacteria, targeting the use of broad-spectrum antibiotics as illustrated in one of the systematic reviews of medical care seems a sensible strategy [29].

### 4.4. Limitations

Publication bias and the possible exclusion of studies in languages other than English are limitations for both the umbrella and systematic review. However, it is unlikely that this would have provided other outcome measures as most studies already used overlapping metrics. Since most studies in the dental systematic review comprised of multifaceted interventions, it is not possible to separate the individual components of the intervention and relate them to a specific metric. The broad range of methodologies in both reviews and heterogeneity of trial designs made it difficult to directly compare the utilisation and choice of specific outcome metrics. Some reported outcomes were subjective such as severity of symptoms, belief in the effectiveness of antibiotics, and thus at risk of reporting bias [22]. Outcome measures that were not considered to be of direct relevance to the aims of the study were also excluded, such as patient and parental knowledge measured by interviews in the patient’s home after the initial consultation [22]. In addition, some reviews included in the umbrella review reported that the included studies were set in western, European countries and US primary care so may not be generalisable to other low- or middle-income countries or settings [22,24,25].

To maximise benefits from implementing an intervention to change prescribing behaviour, interventions need to be tailored to the specific context to target the specific delivery method that is best for the specific group of clinicians [27]. Nonetheless, it may be possible to translate the comprehensive list of outcome measures across primary medical and dental care and to adapt them further across other primary healthcare settings. Assessing effects using randomized intervention studies adhering to an implementation framework would be ideal [58].

## 5. Conclusions

A suite of clinical and patient outcome measures used to evaluate ASPs and interventions in primary medical and dental care has been collated. This will be a useful resource for those selecting appropriate measures to monitor and evaluate the delivery of ASPs in primary healthcare contexts across medicine and dentistry. Employing a mix of clinical and patient outcomes, and quantitative and qualitative measures is recommended. The outcomes selected for measurement should address both delivery of the intended benefits as well as avoidance of negative unintended consequences.

## Figures and Tables

**Table 1:** Characteristics of included studies in the umbrella review across primary medical care.

Study	Objectives	Number of Databases Searched	Published Date Range	Participants	Number, Type	Outcomes Measured
Arroll et al., 2003	To conduct a systematic review of the controlled trials of delayed antibiotic prescription for upper respiratory infections	3	1997–2002	Adults and children	5 studies; 4 randomised controlled trials, 1 controlled before-after study	Reduction in prescriptions consumed or collected Antibiotics consumed by delayed group and immediate group Adverse effects in delayed group compared to the immediate group (diarrhoea) Antibiotic use by intervention delayed group compared to the control group Symptoms in delayed group compared to immediate group Patient satisfaction, beliefs about antibiotics Time off school, distress
de Bont et al., 2015	To review the effectiveness of information leaflets used for informing patients about common infections during consultations in general practice and if this reduces antibiotic use	2	1981–2013	Adult and child patients	8 studies; 1 non-randomised controlled trial, 1 randomised controlled trial, 2 factorial randomised controlled trials; 2 cluster randomised controlled trials, 2 single blinded randomised controlled trials	Antibiotic use Re-consultation rates Severity of symptoms, duration of symptoms, belief in the effectiveness of antibiotics Recall of information Compliance with the prescribed course of antibiotics Patient behaviour (as measured by patient diaries and telephone interviews regarding specific advice given in the consultation after 1 months and 1–2 weeks; telephone questionnaire with the patient or adult (of child patient) after 10–15 days)
Huang et al., 2013	To study the association between family physician use of point-of-care C-reactive protein testing and antibiotic prescribing for respiratory tract infections in general practice	2	1995–2013	Patients with upper and lower respiratory tract infections	13 studies in total: 3 cluster randomised controlled trials, 4 parallel randomised controlled trials, 6 observational studies	Antibiotic prescribing rate Antibiotic prescribing at any time during the 28-day follow-up period Patient satisfaction
Holstiege et al., 2015	To assess the effectiveness of computer-aided clinical decision support systems in improving antibiotic prescribing in primary care	2	2001–2013	Adult and child patients; a wide range of conditions	7 studies in total: 3 cluster randomised trials, 4 randomised controlled trials	Antibiotic prescribing rates Optimal duration of antibiotic prescriptions Prescribing according to guidelines Reduction in antibiotic prescribing Appropriateness of antibiotic treatment
Hu et al., 2016	To analyse the effectiveness of different intervention approaches, targeted different groups (clinicians, parents or both) and whether other factors (study setting, study design and study period), influence effectiveness for reducing antibiotic prescribing for childhood upper respiratory infections.	8	2001–2013	Children aged less than or equal to 18 years diagnosed with any upper respiratory infection	12 studies; 7 cluster randomised controlled trials, 3 non-randomised controlled trials, 2 individual randomised controlled trials	Percentage prescriptions with antibiotic intervention Rate of antimicrobial prescribing per person year Antibiotics prescribing at the index consultation compared to the control group Broad-spectrum prescriptions in intervention compared to control Antibiotic prescribing rate (defined as the number of children who were prescribed one or more antibiotic classes divided by the total number of children assessed for upper respiratory infections during a designated interval) Adjusted antibiotic prescription rate Change in antibiotic prescription rate in intervention compared to control Percentage of patients deciding to use antibiotics Parents administered antibiotics to their children in intervention compared to control Total number of prescriptions in intervention compared to control
Kochling et al., 2018	To summarise the evidence of the effectiveness of interventions in primary care aiming to reduce antibiotic prescriptions in patients for acute respiratory tract infections	2	2006–2016	Primary care physicians and patients greater than or equal to 13 years old	17 studies; 13 cluster randomised controlled studies, 4 randomised controlled trials (at patient level)	Antibiotic prescription rates Absolute number of prescribed antibiotics Absolute reductions of antibiotic prescriptions Antibiotic prescriptions according to guidelines Odds ratios for antibiotic prescriptions Number of adverse events (e.g., hospitalisations, deaths) Number of side effects of Ab therapy Difference in emergency room visits/hospitalisation rates Patient satisfaction Re-consultation rates Return rate visit within 30 days after initial consultation in which no antibiotics were prescribed
Lane et al., 2018	To determine whether locally relevant, real-time syndromic or microbiological infection epidemiology could reduce diagnostic uncertainty and improve antibacterial prescribing.	4	1999–2014	General practitioners, primary care providers, family practice residents, urgent care clinics and community clinics	12 studies; 11 observational studies, 1 prospective cluster randomised controlled trial	Antibacterial prescribing rates Percentage of cases of antibiotics prescribed
O’Sullivan et al., 2016	To assess if written information for patients (or parents of child patients) reduces antibiotic use for acute upper respiratory tract infections in primary care	8	2000 and 2009	Children with upper respiratory tract infections; parents were given written information	2 studies; 2 randomised controlled trials	Antibiotic use Antibiotics used by patients Antibiotics prescribed by clinicians
Saha et al., 2019	To assess the effectiveness of antibiotic stewardship interventions involving pharmacists at improving prescribing by general practitioners	8	1983–2017	General practitioners	35 articles for systematic review; 6 randomised controlled trials, 7 cluster randomised controlled trialss, 19 controlled before-after studies, 2 before-after studies and 1 interrupted time-series	Antibiotic prescribing rate (proportion of all patient visits involving prescription of antibiotics by GPs) Antibiotic adherence rate (proportion of antibiotic prescriptions issued to patients that adhered to guidelines/recommendations) Changes to broad-spectrum antibiotic prescribing rate
Vodicka et al., 2013	To review the effectiveness of educational or behavioural interventions directed to parents, clinicians or both, to reduce antibiotic prescribing for children with respiratory tract infections in primary care	5	1992–2011	Clinicians and parents	17 studies; 12 randomised design, 3 pre–post test, 2 non-randomised	Antibiotic prescribing rates Antibiotics filled per consultation Antibiotics per person-year for children Exceptions to care pathway per 1000 episodes of care Antibiotics/index consultation Antibiotics per upper respiratory infection episode of care Change in annual antibiotics per 100 patient years Antibiotic rates for penicillin, macrolide and cephalosporin Antibiotics per child with acute otitis media Records of incorrect use of antibiotics per all antibiotics Antibiotics per upper respiratory infection Change in proportion of antibiotics<10 days Change in frequency of antibiotics Number of visits with antibiotics for otitis media or sinusitis Mean change in proportion of consultations resulting in antibiotics Antibiotics/respiratory tract infection consultation Appropriate antibiotics/consultations Incorrect antibiotic order compared to all antibiotic orders Antibiotic adherence to guidelines compared to total antibiotics Proportion/number of consultations resulting in antibiotics Appropriate antibiotics/consultations Antibiotic in adherence to guidelines Antibiotics/respiratory tract infection consultation Adverse events Re-consultation rates

**Table 2:** Characteristics of included studies in the systematic review across primary dental care.

Article	Location	Years Study/Timing	Setting	Study Design	Participants	Objective	Outcomes Measured
Chate et al., 2006	England	2002–2004	General dental practices	Pre–post	Dentists	To use the intervention of education and prescribing guidelines to reduce the number of antibiotics inappropriately prescribed by general dental practitioners, and to increase overall prescription accuracy.	Number of antibiotics according to guidelines (dose, frequency and duration) Numbers of antibiotics prescribed for specific clinical indications Numbers of prophylactic antibiotics prescribed for specific medical conditions
Elouaflaoui et al., 2016	Scotland	2012–2013	General dental practices	Prospective; 1 control group and 2 intervention groups	Dentists in NHS practices in Scotland	To compare the effectiveness of individualised audit and feedback interventions for the translation into practice of national guidance recommendations on antibiotic prescribing. A secondary objective was to explore dentists’ experiences of and responses to the individualised A&F interventions and to increase understanding of the factors associated dental antibiotic prescribing.	Antibiotic prescribing rate: Primary outcome: Total number of claims Total number and DDD of antibiotics Total number and DDD of amoxicillin 3g sachets Total number and DDD of broad-spectrum antibiotics Secondary outcomes: Total DDD of antibiotics/100 claims Total number of amoxicillin 3g sachets/100 claims Total DDD of amoxicillin 3g sachets/100 claims Total number of broad-spectrum antibiotics/100 claims Total DDD of broad-spectrum antibiotics/100 claims
Palmer et al., 2001	England	Could not find date	General dental practices	Pre–post	Dentists	To investigate if clinical audit, with an intervention of education and guidelines, can improve general dental practitioners’ antibiotic prescribing	Total numbers of antibiotics Numbers of antibiotic divided by type Numbers of antibiotics prescribed for specific medical conditions Numbers of antibiotics prescribed for specific clinical indications Number of antibiotics according to guidelines (dose, frequency and duration)
Seager et al., 2006	Wales	Could not find date	Primary care general dental practices	Randomised controlled trial	Dentists	To investigate if guidelines, or guidelines and education will improve antibiotic prescribing compared to a control group.	Number and percentage of antibiotic prescriptions for patients with dental pain Number and percentage of inappropriate antibiotic prescriptions (defined as the provision of an antibiotic to a patient who did not present with a symptom indicative of spreading infection)
Teoh et al., 2020	Melbourne, Australia	2019	General dental practices	Pre–post	Dentists	The aim of this pilot study was to evaluate a multimodal intervention to improve dental prescribing. The intervention comprised two parts: Targeted education about drug use in dentistry and an online prescribing tool.	Number of antibiotic prescriptions before and after the intervention Number of inappropriate indications for antibiotic prescription before and after the intervention Accuracy of the prescriptions according to the Australian therapeutic guidelines before and after the intervention Confidence and attitude of practitioners towards the online dental prescribing tool.

**Table 3:** Comparing outcomes between primary medical and dental care to evaluate.

Outcome Measures	Primary Medical Care	Primary Dental Care
Quantity of antibiotic use	Absolute numbers of prescribed antibiotics (in intervention compared to control groups) Antibiotics used by patients Antibiotic use (odds ratios) Percentage of patients deciding to use antibiotics Reduction in prescribing/prescriptions consumed or collected	Numbers of antibiotics (total, type) Number of antibiotic prescriptions before and after the intervention
Rate of antibiotic use	Antibiotic prescribing rate (total, type, by consultation, by patient, per person-year) Antibiotic prescribing at the index consultation compared to the control group Change in antibiotic prescription rate in intervention compared to control group Changes to broad-spectrum antibiotic prescribing rate Change in proportion/frequency of antibiotics	Antibiotic prescribing rate using/100 claims Number/percentage of antibiotics per dental pain visit Total number of claims/100 claims Total DDD of antibiotics/100 claims (total, type) Total number of broad-spectrum antibiotics/100 claims Total DDD of broad-spectrum antibiotics/100 claims
Quality of antibiotic use (appropriateness, appropriate indications and accuracy of prescriptions according to guidelines)	Antibiotics prescribed in adherence to guidelines Antibiotic adherence rate/patient compliance (proportion of antibiotic prescriptions that adhered to guidelines/recommendations) Appropriateness of antibiotic treatment (indications) Broad-spectrum prescriptions in intervention compared to control Optimal duration of antibiotic prescriptions Records of incorrect use of antibiotics per all antibiotics	Number of antibiotics according to guidelines (dose, frequency and duration) Number/percentage of inappropriate indications for antibiotic prescription Numbers of antibiotics prescribed for specific medical/clinical indications
Confidence of clinicians towards prescribing		Confidence and attitude of practitioners towards the online dental prescribing tool.
Consultation/Re-consultation rates	Appropriate antibiotics/consultations (proportion, number) Antibiotic prescribing at any time during a specified follow-up period Number of visits with antibiotics for specific clinical conditions Mean change in proportion of consultations resulting in antibiotics Re-consultation rates/return rate visit	
Adverse effects	Number of adverse effects relating to antibiotic use Number of adverse events (e.g., emergency room visits, hospitalisations, deaths) Time off school, distress	
Severity of symptoms	Presence, severity and duration of symptoms, belief in the effectiveness of antibiotics	
Other patient outcomes	Patient satisfaction Patient behaviour	

## Supplementary Materials

The following are available online at https://www.mdpi.com/2079-6382/9/9/607/s1. S1: Example of the search strategy used to identify relevant papers for the umbrella review across primary medical care, S2: Example of the search strategy used to identify relevant papers for the systematic review across primary dental care, Table S1: Methodological quality assessment of systematic reviews included in the umbrella review across primary medical care using the Critical Skills Appraisal Programme (CASP) checklist for systematic reviews, Table S2: Methodological quality assessment of studies included in the systematic review across primary dental care using the Quality Assessment Tool for Studies with Diverse Design (QATSSD), Table S3: Mapping the ten systematic reviews of the umbrella review to their 111 constituent primary research studies.
