# Supplementary material for: Measuring Antibiotic Stewardship Programmes and Initiatives: An Umbrella Review in Primary Care Medicine and a Systematic Review of Dentistry
Source: Antibiotics (Basel). 2020 Sep 16;9(9):607. doi: 10.3390/antibiotics9090607 (PMC7558917; doi:10.3390/antibiotics9090607)
Supplement: Supplementary file 1 [file antibiotics-09-00607-s001.pdf]

## Supplementary Material

**S1. Example of the search strategy used to identify relevant papers for the umbrella review across primary medical care**

1. antimicrobial steward* mp. [mp=title, abstract, original title, name of substance word, subject heading word, floating sub-heading word, keyword heading word, organism supplementary concept word, protocol supplementary concept word, rare disease supplementary concept word, unique identifier, synonyms]

2. antibiotic steward* mp. [mp=title, abstract, original title, name of substance word, subject heading word, floating sub-heading word, keyword heading word, organism supplementary concept word, protocol supplementary concept word, rare disease supplementary concept word, unique identifier, synonyms]

3. antibiotic resistance mp. [mp=title, abstract, original title, name of substance word, subject heading word, floating sub-heading word, keyword heading word, organism supplementary concept word, protocol supplementary concept word, rare disease supplementary concept word, unique identifier, synonyms]

4. "antibiotic use" mp. [mp=title, abstract, original title, name of substance word, subject heading word, floating sub-heading word, keyword heading word, organism supplementary concept word, protocol supplementary concept word, rare disease supplementary concept word, unique identifier, synonyms]

5. antimicrobial mp. [mp=title, abstract, original title, name of substance word, subject heading word, floating sub-heading word, keyword heading word, organism supplementary concept word, protocol supplementary concept word, rare disease supplementary concept word, unique identifier, synonyms]

6. antibiotic resistance mp. [mp=title, abstract, original title, name of substance word, subject heading word, floating sub-heading word, keyword heading word, organism supplementary concept word, protocol supplementary concept word, rare disease supplementary concept word, unique identifier, synonyms]

7. 1 or 2 or 3 or 4 or 5 or 6

8. primary medical care mp. [mp=title, abstract, original title, name of substance word, subject heading word, floating sub-heading word, keyword heading word, organism supplementary concept word, protocol supplementary concept word, rare disease supplementary concept word, unique identifier, synonyms]

9. primary care mp. [mp=title, abstract, original title, name of substance word, subject heading word, floating sub-heading word, keyword heading word, organism supplementary concept word, protocol supplementary concept word, rare disease supplementary concept word, unique identifier, synonyms]

10. primary health care mp. [mp=title, abstract, original title, name of substance word, subject heading word, floating sub-heading word, keyword heading word, organism supplementary concept word, protocol supplementary concept word, rare disease supplementary concept word, unique identifier, synonyms]

11. primary healthcare mp. [mp=title, abstract, original title, name of substance word, subject heading word, floating sub-heading word, keyword heading word, organism supplementary concept word, protocol supplementary concept word, rare disease supplementary concept word, unique identifier, synonyms]

12. general practi* mp. [mp=title, abstract, original title, name of substance word, subject heading word, floating sub-heading word, keyword heading word, organism supplementary concept word, protocol supplementary concept word, rare disease supplementary concept word, unique identifier, synonyms]

13. outpatient mp. [mp=title, abstract, original title, name of substance word, subject heading word, floating sub-heading word, keyword heading word, organism supplementary concept word, protocol supplementary concept word, rare disease supplementary concept word, unique identifier, synonyms]

14. medical mp. [mp=title, abstract, original title, name of substance word, subject heading word, floating sub-heading word, keyword heading word, organism supplementary concept word, protocol supplementary concept word, rare disease supplementary concept word, unique identifier, synonyms]

15. 8 or 9 or 10 or 11 or 12 or 13 or 14

16. systematic review*.mp. [mp=title, abstract, original title, name of substance word, subject heading word, floating sub-heading word, keyword heading word, organism supplementary concept word, protocol supplementary concept word, rare disease supplementary concept word, unique identifier, synonyms]

17. 7 and 14 and 16

**S2. Example of the search strategy used to identify relevant papers for the systematic review across primary dental care**

1. antimicrobial steward* mp. [mp=title, abstract, original title, name of substance word, subject heading word, floating sub-heading word, keyword heading word, organism supplementary concept word, protocol supplementary concept word, rare disease supplementary concept word, unique identifier, synonyms]

2. antibiotic steward* mp. [mp=title, abstract, original title, name of substance word, subject heading word, floating sub-heading word, keyword heading word, organism supplementary concept word, protocol supplementary concept word, rare disease supplementary concept word, unique identifier, synonyms]

3. antibiotic resistance mp. [mp=title, abstract, original title, name of substance word, subject heading word, floating sub-heading word, keyword heading word, organism supplementary concept word, protocol supplementary concept word, rare disease supplementary concept word, unique identifier, synonyms]

4. “antibiotic use” mp. [mp=title, abstract, original title, name of substance word, subject heading word, floating sub-heading word, keyword heading word, organism supplementary concept word, protocol supplementary concept word, rare disease supplementary concept word, unique identifier, synonyms]

5. antimicrobial mp. [mp=title, abstract, original title, name of substance word, subject heading word, floating sub-heading word, keyword heading word, organism supplementary concept word, protocol supplementary concept word, rare disease supplementary concept word, unique identifier, synonyms]

6. antibiotic resistance mp. [mp=title, abstract, original title, name of substance word, subject heading word, floating sub-heading word, keyword heading word, organism supplementary concept word, protocol supplementary concept word, rare disease supplementary concept word, unique identifier, synonyms]

7. 1 or 2 or 3 or 4 or 5 or 6

8. dental mp. [mp=title, abstract, original title, name of substance word, subject heading word, floating sub-heading word, keyword heading word, organism supplementary concept word, protocol supplementary concept word, rare disease supplementary concept word, unique identifier, synonyms]
9. oral health mp. [mp=title, abstract, original title, name of substance word, subject heading word, floating sub-heading word, keyword heading word, organism supplementary concept word, protocol supplementary concept word, rare disease supplementary concept word, unique identifier, synonyms]
10. dentist* mp. [mp=title, abstract, original title, name of substance word, subject heading word, floating sub-heading word, keyword heading word, organism supplementary concept word, protocol supplementary concept word, rare disease supplementary concept word, unique identifier, synonyms]
11. 8 or 9 or 10

12. intervention* mp. [mp=title, abstract, original title, name of substance word, subject heading word, floating sub-heading word, keyword heading word, organism supplementary concept word, protocol supplementary concept word, rare disease supplementary concept word, unique identifier, synonyms]
13. audit mp. [mp=title, abstract, original title, name of substance word, subject heading word, floating sub-heading word, keyword heading word, organism supplementary concept word, protocol supplementary concept word, rare disease supplementary concept word, unique identifier, synonyms]
14. outreach mp. [mp=title, abstract, original title, name of substance word, subject heading word, floating sub-heading word, keyword heading word, organism supplementary concept word, protocol supplementary concept word, rare disease supplementary concept word, unique identifier, synonyms]
15. trial* mp. [mp=title, abstract, original title, name of substance word, subject heading word, floating sub-heading word, keyword heading word, organism supplementary concept word, protocol supplementary concept word, rare disease supplementary concept word, unique identifier, synonyms]

16. 12 and 13 and 14 and 15

17. 7 and 11 and 16

**Table S1. Methodological quality assessment of systematic reviews included in the umbrella review across primary medical care using the Critical Skills Appraisal Programme (CASP) checklist for systematic reviews.**

| | **A) Are the results of the review valid?** | | | | | **B) What are the results?** | | **C) Will the results help locally?** | | |
|---|---|---|---|---|---|---|---|---|---|---|
| | Clear question? | Right type of papers? | All relevant studies included? | QA of included studies | Reasonable to combine results? | Overall results | Precision? | Applicability of results | All important outcomes? | Are the benefits worth the harms and costs? |
| Arroll et al, 2003 | Yes | Yes | Yes | Yes | Yes | Yes | Yes | Yes | Yes | Yes |
| de Bont et al, 2015 | Yes | Yes | Yes | Yes | No | Yes | Yes | Yes | Yes | Yes |

| | | | | | | | | | | |
|---|---|---|---|---|---|---|---|---|---|---|
| Huang et al, 2013 | Yes | Yes | Yes | Yes | Can't tell | Yes | Yes | Yes | Yes | Yes |
| Holsteige et al, 2015 | Yes | Yes | Yes | Yes | No | Yes | Can't tell | Yes | Yes | Yes |
| Hu et al, 2016 | Yes | Yes | Yes | Yes | Can't tell | Yes | Can't tell | Yes | Yes | Yes |
| Kochling et al, 2018 | Yes | Yes | Yes | Yes | Can't tell | Yes | Can't tell | Yes | Yes | Yes |
| Lane et al, 2018 | Yes | Yes | Yes | Yes | Can't tell | Can't tell | Can't tell | Yes | Yes | Yes |
| O'Sullivan et al, 2016 | Yes | Yes | Yes | Yes | No | Yes | Yes | Yes | Yes | Yes |
| Saha et al, 2019 | Yes | Yes | Yes | Yes | Can't tell | Yes | Yes | Yes | Yes | Yes |
| Vodicka et al, 2013 | Yes | Yes | Yes | Yes | Can't tell | Yes | Can't tell | Yes | Yes | Yes |

**Table S2. Methodological quality assessment of studies included in the systematic review across primary dental care using the Quality Assessment Tool for Studies with Diverse Design (QATSSD).** The second mark relates to the scores of the second marker who undertook a parallel assessment of all studies in the systematic review.

| | 1. Explicit theoretical framework | 2. Statement of aims/ objectives in main body of report | 3. Clear description of research setting | 4. Evidence of sample size considered in terms of analysis | 5. Representative sample of target group of a reasonable size | 6. Description of procedure for data collection | 7. Rationale for choice of data collection tool(s) | 8. Detailed recruitment data | 9. Statistical assessment of reliability and validity of measurement tool(s) |
|---|---|---|---|---|---|---|---|---|---|
| Chate et al, 2006 | 2 or 1 | 3 and 3 | 3 and 2 | 0 and 1 | 2 and 2 | 2 and 2 | 0 and 2 | 2 and 1 | 2 and 0 |
| Elouaflaoui et al, 2016 | 3 and 3 | 3 and 3 | 3 and 3 | 3 and 3 | 3 and 3 | 3 and 3 | 2 and 2 | 3 and 3 | 3 and 2 |
| Palmer et al, 2001 | 3 and 3 | 3 and 3 | 3 and 3 | 2 and 0 | 1 and 3 | 3 and 3 | 1 and 1 | 2 and 1 | 1 and 1 |
| Seager et al, 2006 | 3 and 3 | 3 and 3 | 3 and 3 | 3 and 3 | 3 and 3 | 3 and 3 | 1 and 2 | 3 and 3 | 2 and 1 |
| Teoh et al, 2020 | 2 and 2 | 3 and 3 | 3 and 2 | 3 and 3 | 2 and 3 | 3 and 3 | 1 and 1 | 2 and 3 | 1 and 1 |
| Zahabiyoun et al, 2015 | 3 and 2 | 3 and 3 | 1 and 1 | 0 and 0 | 0 and 0 | 2 and 2 | 1 and 1 | 1 and 1 | 1 and 0 |

**Table S2 (continued)**

| | 10. Fit between stated research question and method of data collection | 11. Fit between stated research question and format and content of data collection tool e.g. interview schedule | 12. Fit between research question and method of analysis | 13. Good justification for analytical method selected | 14. Assessment of reliability of analytical process | 15. Evidence of user involvement in design | 16. Strengths and limitations critically discussed | Total | % Total | Average total |
|---|---|---|---|---|---|---|---|---|---|---|
| Chate et al, 2006 | 3 and 3 | 0 and 0 | 3 and 3 | 2 and 2 | 0 and 0 | 3 and 0 | 1 and 1 | 28 and 23 | 67 and 55 | 61 |
| Elouaflaoui et al, 2016 | 3 and 3 | 2 and 0 | 3 and 3 | 3 and 2 | 3 and 0 | 3 and 2 | 3 and 3 | 46 and 38 | 96 and 90 | 93 |
| Palmer et al, 2001 | 3 and 3 | 0 and 0 | 3 and 3 | 2 and 2 | 0 and 0 | 1 and 0 | 0 and 1 | 28 and 27 | 67 and 64 | 66 |
| Seager et al, 2006 | 3 and 3 | 0 and 0 | 3 and 3 | 3 and 3 | 0 and 0 | 2 and 3 | 2 and 1 | 37 and 37 | 88 and 88 | 88 |
| Teoh et al, 2020 | 3 and 3 | 2 and 0 | 3 and 3 | 2 and 2 | 2 and 0 | 1 and 0 | 2 and 3 | 35 and 32 | 73 and 75 | 74 |
| Zahabiyoun et al, 2015 | 0 and 0 | 0 and 0 | 3 and 3 | 2 and 1 | 0 and 0 | 1 and 0 | 1 and 1 | 19 and 15 | 45 and 36 | 41 |

**Table S3 Mapping the ten systematic reviews of the umbrella review to their 111 constituent primary research studies from which metrics used to assess success of AMS interventions originated**

| | Systematic reviews of the umbrella review across medical care | | | | | | | | | |
|---|---|---|---|---|---|---|---|---|---|---|
| **Primary research study** | Arroll et al, 2003 | de Bont et al, 2015 | Holsteige et al, 2014 | Hu et al, 2016 | Huang et al, 2013 | Kochling et al, 2018 | Lane et al, 2018 | O'Sullivan et al, 2016 | Saha et al, 2019 | Vodicka et al, 2013 |
| Agnew et al, 2013[1] | | ✓ | | | | | | | | |
| Altiner et al, 2007[2] | | | | | | ✓ | | | | |
| Andreeva et al, 2014[3] | | | | | | ✓ | | | | |
| Arroll et al, 2002[4] | ✓ | | | | | | | | | |
| Ashe et al, 2006[5] | | | | | | | | | | ✓ |
| Avorn et al, 1983[6] | | | | | | | | | ✓ | |
| Bauchner et al, 2006[7] | | | | | | | | | | ✓ |
| Bjerrum et al, 2004[8] | | | | | ✓ | | | | | |
| Bjerrum et al, 2006[9] | | | | | | ✓ | | | | |
| Bourgeois et al, 2010[10] | | | ✓ | | | | | | | ✓ |
| Braybrook et al, 1996[11] | | | | | | | | | ✓ | |
| Briel et al, 2006[12] | | | | | | ✓ | | | | |
| Cals et al, 2009[13] | | | | | ✓ | ✓ | | | | |
| Cals et al, 2010[14] | | | | | ✓ | ✓ | | | | |
| Cals et al, 2011[15] | | | | | ✓ | | | | | |
| Cals et al, 2013[16] | | | | | ✓ | | | | | |
| Cates et al, 1999[17] | ✓ | | | | | | | | | |
| Christakis et al, 2001[18] | | | ✓ | | | | | | | ✓ |
| Coenen et al, 2004[19] | | | | | | | | | ✓ | |
| Cohen et al, 2000[20] | | | | | | | | | | ✓ |
| Daniel et al, 2005[21] | | | | | | | ✓ | | | |
| Davis et al, 2007[22] | | | ✓ | | | | | | | |
| De Santis et al, 1994[23] | | | | | | | | | ✓ | |
| Diederichsen et al, 2000[24] | | | | | ✓ | | | | | |
| Dowell et al, 2001[25] | ✓ | | | | | | | | | |
| Doyne et al, 2004[26] | | | | | | | | | | ✓ |
| Ekedahl et al, 1995[27] | | | | | | | | | ✓ | |

| | | | | | | | | | | |
|---|---|---|---|---|---|---|---|---|---|---|
| Esmaily et al, 2010[28] | | | | ✓ | | | | | ✓ | |
| Everitt et al, 2006[29] | | ✓ | | | | | | | | |
| Fagan MS, 2001[30] | | | | | ✓ | | | | | |
| Finkelstein et al, 2001[31] | | | | ✓ | | | | | | ✓ |
| Forrest et al, 2013[32] | | | ✓ | | | | | | | |
| Francis et al, 2006[33] | | | | | | | | | | ✓ |
| Francis et al, 2009[34] | | ✓ | | ✓ | | | | ✓ | | ✓ |
| Gauld VA, 1981[35] | | ✓ | | | | | | | | |
| Gerber et al, 2013[36] | | | | ✓ | | | | | | |
| Gesteland et al, 2007[37] | | | | | | | ✓ | | | |
| Gjelstad et al, 2013[38] | | | | ✓ | ✓ | | | | | |
| Gonzales et al, 2005[39] | | | | ✓ | | | | | | |
| Gonzales et al, 2011[40] | | | | | ✓ | | | | | |
| Gonzales et al, 2013[41] | | | ✓ | | ✓ | | | | | |
| Gonzalez et al, 2008[42] | | | | | | | ✓ | | | |
| Gulliford et al, 2014[43] | | | | | | ✓ | | | | |
| Hall et al, 2001[44] | | | | | | | | | ✓ | |
| Hebert et al, 2012[45] | | | | | | | ✓ | | | |
| Hernandez-Santiago et al, 2015[46] | | | | | | | | | ✓ | |
| Hickman et al, 2003[47] | | | | | | | | | ✓ | |
| Hui Min Lee et al, 2017[48] | | | | | | ✓ | | | | |
| Ilett et al, 2000[49] | | | | | | | | | ✓ | |
| Ives et al, 1987[50] | | | | | | | | | ✓ | |
| Jakobsen et al, 2010[51] | | | | | ✓ | | | | | |
| Jones et al, 2004[52] | | | | | | | ✓ | | | |
| Juzych et al, 2005[53] | | | | ✓ | | | | | ✓ | ✓ |
| Kavanagh et al, 2011[54] | | | | | ✓ | | | | | |
| Lagerløv et al, 2000[55] | | | | | | | | | ✓ | |
| Legare et al, 2012[56] | | | | ✓ | | | | | | |
| Linder et al, 2009[57] | | | ✓ | | | ✓ | | | | |
| Linder et al, 2010[58] | | | | | | ✓ | | | | |
| Little et al, 1997[59] | ✓ | | | | | | | | | |

| | | | | | | | | | | |
|---|---|---|---|---|---|---|---|---|---|---|
| Little et al, 2001[60] | ✓ | | | | | | | | | |
| Little et al, 2005[61] | | ✓ | | | | | | | | |
| Little et al, 2013[62] | | | | | | ✓ | | | | |
| Llor et al, 2011[63] | | | | | | ✓ | | | | |
| Llor et al, 2012a[64] | | | | | ✓ | | | | | |
| Llor et al, 2012b[65] | | | | | ✓ | | | | | |
| Lundborg et al, 1997[66] | | | | | | | | | ✓ | |
| Lundborg et al, 1999[67] | | | | | | | | | ✓ | |
| Macfarlane et al, 1997[68] | | ✓ | | | | | | | | |
| Macfarlane et al, 2002[69] | | ✓ | | | | | | | | |
| Madridejos-Mora et al, 2004[70] | | | | | | | | | ✓ | |
| Magrini et al, 2014[71] | | | | | | | | | ✓ | |
| Mainous et al, 2000[72] | | | | | | | | ✓ | | ✓ |
| Margolis et al, 1992[73] | | | | | | | | | | ✓ |
| Martens et al, 2006[74] | | | | | | | | | ✓ | |
| McGinn et al, 2013[75] | | | ✓ | | | ✓ | | | | |
| Meeker et al, 2016[76] | | | | | | ✓ | | | | |
| Melbye et al, 1995[77] | | | | | ✓ | | | | | |
| Naughton et al, 2009[78] | | | | | | | | | ✓ | |
| Ndefo et al, 2017[79] | | | | | | | | | ✓ | |
| Olson et al, 2011[80] | | | | | | | ✓ | | | |
| Peterson et al, 1997[81] | | | | | | | | | ✓ | |
| Price et al, 2014[82] | | | | | | | ✓ | | | |
| Pshetizky et al, 2003[83] | | | | ✓ | | | | | | |
| Razon et al, 2005[84] | | | | | | | | | | ✓ |
| Regev-Yochay et al, 2011[85] | | | | ✓ | | | | | | ✓ |
| Roque et al, 2016[86] | | | | | | | | | ✓ | |
| Saint et al, 1999[87] | | | | | | | | | ✓ | |
| Santoso et al, 1996[88] | | | | | | | | | ✓ | |
| Schaffner et al,1983[89] | | | | | | | | | ✓ | |
| Shah et al, 2014[90] | | | | | | | ✓ | | | |
| Simonsen et al, 2011[91] | | | | | | | ✓ | | | |

| | | | | | | | | | | |
|---|---|---|---|---|---|---|---|---|---|---|
| Smabrekke et al, 2002[92] | | | | | | | | | | ✓ |
| Smeets et al, 2009[93] | | | | | | | | | ✓ | |
| Stewart et al, 2000[94] | | | | | | | | | ✓ | |
| Sustersic et al, 2013[95] | | ✓ | | | | | | | | |
| Taylor et al, 2005[96] | | | | ✓ | | | | | | ✓ |
| Temte et al, 1999[97] | | | | | | | ✓ | | | |
| Van Driel et al, 2007[98] | | | | | | | | | ✓ | |
| Vellinga et al, 2016[99] | | | | | | | | | ✓ | |
| Veninga et al, 2000[100] | | | | | | | | | ✓ | |
| Vervloet et al, 2016[101] | | | | | | | | | ✓ | |
| Vinnard et al, 2013[102] | | | | | | | | | ✓ | |
| Weiss et al, 2011[103] | | | | | | | | | ✓ | |
| Welschen et al, 2004[104] | | | | | | | | | ✓ | |
| Wheeler et al, 2001[105] | | | | ✓ | | | | | | |
| Wilf-Miron et al, 2012[106] | | | | | | | | | ✓ | |
| Wilson et al, 2003[107] | | | | | | | | | | ✓ |
| Worrall et al, 2010[108] | | | | | | ✓ | | | | |
| Zelicoff et al, 2001[109] | | | | | | | ✓ | | | |

## References


1. Agnew J, Taaffe M, Darker C, O'Shea B, Clarke J. Delayed prescribing of antibiotics for respiratory tract infections: use of information leaflets. Ir Med J. 2013;106(8):243-4.
2. Altiner A, Brockmann S, Sielk M, Wilm S, Wegscheider K, Abholz HH. Reducing antibiotic prescriptions for acute cough by motivating GPs to change their attitudes to communication and empowering patients: a cluster-randomized intervention study. J Antimicrob Chemother. 2007;60(3):638-44.
3. Andreeva E, Melbye H. Usefulness of C-reactive protein testing in acute cough/respiratory tract infection: an open cluster-randomized clinical trial with C-reactive protein testing in the intervention group. BMC Fam Pract. 2014;15:80.
4. Arroll B, Kenealy T, Kerse N. Do delayed prescriptions reduce the use of antibiotics for the common cold? A single-blind controlled trial. J Fam Pract. 2002;51(4):324-8.
5. Ashe D, Patrick PA, Stempel MM, Shi Q, Brand DA. Educational posters to reduce antibiotic use. J Pediatr Health Care. 2006;20(3):192-7.
6. Avorn J, Soumerai SB. Improving drug-therapy decisions through educational outreach. A randomized controlled trial of academically based "detailing". N Engl J Med. 1983;308(24):1457-63.

7. Bauchner H, Marchant CD, Bisbee A, Heeren T, Wang B, McCabe M, et al. Effectiveness of Centers for Disease Control and Prevention recommendations for outcomes of acute otitis media. Pediatrics. 2006;117(4):1009-17.
8. Bjerrum L, Gahrn-Hansen B, Munck AP. C-reactive protein measurement in general practice may lead to lower antibiotic prescribing for sinusitis. Br J Gen Pract. 2004;54(506):659-62.
9. Bjerrum L, Cots JM, Llor C, Molist N, Munck A. Effect of intervention promoting a reduction in antibiotic prescribing by improvement of diagnostic procedures: a prospective, before and after study in general practice. Eur J Clin Pharmacol. 2006;62(11):913-8.
10. Bourgeois FC, Linder J, Johnson SA, Co JP, Fiskio J, Ferris TG. Impact of a computerized template on antibiotic prescribing for acute respiratory infections in children and adolescents. Clin Pediatr (Phila). 2010;49(10):976-83.
11. Braybrook S, Walker R. Influencing prescribing in primary care: a comparison of two different prescribing feedback methods. J Clin Pharm Ther. 1996;21(4):247-54.
12. Briel M, Langewitz W, Tschudi P, Young J, Hugenschmidt C, Bucher HC. Communication training and antibiotic use in acute respiratory tract infections. A cluster randomised controlled trial in general practice. Swiss Med Wkly. 2006;136(15-16):241-7.
13. Cals JW, Butler CC, Hopstaken RM, Hood K, Dinant GJ. Effect of point of care testing for C reactive protein and training in communication skills on antibiotic use in lower respiratory tract infections: cluster randomised trial. BMJ. 2009;338:b1374.
14. Cals JW, Schot MJ, de Jong SA, Dinant GJ, Hopstaken RM. Point-of-care C-reactive protein testing and antibiotic prescribing for respiratory tract infections: a randomized controlled trial. Ann Fam Med. 2010;8(2):124-33.
15. Cals JW, Ament AJ, Hood K, Butler CC, Hopstaken RM, Wassink GF, et al. C-reactive protein point of care testing and physician communication skills training for lower respiratory tract infections in general practice: economic evaluation of a cluster randomized trial. J Eval Clin Pract. 2011;17(6):1059-69.
16. Cals JW, de Bock L, Beckers PJ, Francis NA, Hopstaken RM, Hood K, et al. Enhanced communication skills and C-reactive protein point-of-care testing for respiratory tract infection: 3.5-year follow-up of a cluster randomized trial. Ann Fam Med. 2013;11(2):157-64.
17. Cates C. An evidence based approach to reducing antibiotic use in children with acute otitis media: controlled before and after study. BMJ. 1999;318(7185):715-6.
18. Christakis DA, Zimmerman FJ, Wright JA, Garrison MM, Rivara FP, Davis RL. A randomized controlled trial of point-of-care evidence to improve the antibiotic prescribing practices for otitis media in children. Pediatrics. 2001;107(2):E15.
19. Coenen S, Van Royen P, Michiels B, Denekens J. Optimizing antibiotic prescribing for acute cough in general practice: a cluster-randomized controlled trial. J Antimicrob Chemother. 2004;54(3):661-72.
20. Cohen R AF, Callens A et al. Medico-economic evaluation of an educational intervention to optimize children uncomplicated nasopharyngitis treatment in ambulatory care. Med Mal Infect. 2000;30:691-8.
21. Daniel JB, Heisey-Grove D, Gadam P, Yih W, Mandl K, Demaria A, Jr., et al. Connecting health departments and providers: syndromic surveillance's last mile. MMWR Suppl. 2005;54:147-50.
22. Davis RL, Wright J, Chalmers F, Levenson L, Brown JC, Lozano P, et al. A cluster randomized clinical trial to improve prescribing patterns in ambulatory pediatrics. PLoS Clin Trials. 2007;2(5):e25.
23. De Santis G, Harvey KJ, Howard D, Mashford ML, Moulds RF. Improving the quality of antibiotic prescription patterns in general practice. The role of educational intervention. Med J Aust. 1994;160(8):502-5.

24. Diederichsen HZ, Skamling M, Diederichsen A, Grinsted P, Antonsen S, Petersen PH, et al. Randomised controlled trial of CRP rapid test as a guide to treatment of respiratory infections in general practice. Scand J Prim Health Care. 2000;18(1):39-43.
25. Dowell J, Pitkethly M, Bain J, Martin S. A randomised controlled trial of delayed antibiotic prescribing as a strategy for managing uncomplicated respiratory tract infection in primary care. Br J Gen Pract. 2001;51(464):200-5.
26. Doyne EO, Alfaro MP, Siegel RM, Atherton HD, Schoettker PJ, Bernier J, et al. A randomized controlled trial to change antibiotic prescribing patterns in a community. Arch Pediatr Adolesc Med. 2004;158(6):577-83.
27. Ekedahl A, Andersson SI, Hovelius B, Molstad S, Liedholm H, Melander A. Drug prescription attitudes and behaviour of general practitioners. Effects of a problem-oriented educational programme. Eur J Clin Pharmacol. 1995;47(5):381-7.
28. Esmaily HM, Silver I, Shiva S, Gargani A, Maleki-Dizaji N, Al-Maniri A, et al. Can rational prescribing be improved by an outcome-based educational approach? A randomized trial completed in Iran. J Contin Educ Health Prof. 2010;30(1):11-8.
29. Everitt HA, Little PS, Smith PW. A randomised controlled trial of management strategies for acute infective conjunctivitis in general practice. BMJ. 2006;333(7563):321.
30. Fagan MS. [Can use of antibiotics in acute bronchitis be reduced?]. Tidsskr Nor Laegeforen. 2001;121(4):455-8.
31. Finkelstein JA, Davis RL, Dowell SF, Metlay JP, Soumerai SB, Rifas-Shiman SL, et al. Reducing antibiotic use in children: a randomized trial in 12 practices. Pediatrics. 2001;108(1):1-7.
32. Forrest CB, Fiks AG, Bailey LC, Localio R, Grundmeier RW, Richards T, et al. Improving adherence to otitis media guidelines with clinical decision support and physician feedback. Pediatrics. 2013;131(4):e1071-81.
33. Francis DO, Beckman H, Chamberlain J, Partridge G, Greene RA. Introducing a multifaceted intervention to improve the management of otitis media: how do pediatricians, internists, and family physicians respond? Am J Med Qual. 2006;21(2):134-43.
34. Francis NA, Butler CC, Hood K, Simpson S, Wood F, Nuttall J. Effect of using an interactive booklet about childhood respiratory tract infections in primary care consultations on reconsulting and antibiotic prescribing: a cluster randomised controlled trial. BMJ. 2009;339:b2885.
35. Gauld VA. Written advice: compliance and recall. J R Coll Gen Pract. 1981;31(230):553-6.
36. Gerber JS, Prasad PA, Fiks AG, Localio AR, Grundmeier RW, Bell LM, et al. Effect of an outpatient antimicrobial stewardship intervention on broad-spectrum antibiotic prescribing by primary care pediatricians: a randomized trial. JAMA. 2013;309(22):2345-52.
37. Gesteland PH, Samore MH, Pavia AT, Srivastava R, Korgenski K, Gerber K, et al. Informing the front line about common respiratory viral epidemics. AMIA Annu Symp Proc. 2007:274-8.
38. Gjelstad S, Hoye S, Straand J, Brekke M, Dalen I, Lindbaek M. Improving antibiotic prescribing in acute respiratory tract infections: cluster randomised trial from Norwegian general practice (prescription peer academic detailing (Rx-PAD) study). BMJ. 2013;347:f4403.
39. Gonzales R, Corbett KK, Leeman-Castillo BA, Glazner J, Erbacher K, Darr CA, et al. The "minimizing antibiotic resistance in Colorado" project: impact of patient education in improving antibiotic use in private office practices. Health Serv Res. 2005;40(1):101-16.
40. Gonzales R, Aagaard EM, Camargo CA, Jr., Ma OJ, Plautz M, Maselli JH, et al. C-reactive protein testing does not decrease antibiotic use for acute cough illness when compared to a clinical algorithm. J Emerg Med. 2011;41(1):1-7.
41. Gonzales R, Anderer T, McCulloch CE, Maselli JH, Bloom FJ, Jr., Graf TR, et al. A cluster randomized trial of decision support strategies for reducing antibiotic use in acute bronchitis. JAMA Intern Med. 2013;173(4):267-73.
42. Gonzalez Moran F MCI, Vanaclocha H. Real time information. A necessary tool in epidemiological surveillance. Gra Sanit. 2008;22:162-7.

43. Gulliford MC, van Staa T, Dregan A, McDermott L, McCann G, Ashworth M, et al. Electronic health records for intervention research: a cluster randomized trial to reduce antibiotic prescribing in primary care (eCRT study). Ann Fam Med. 2014;12(4):344-51.
44. Hall L, Eccles M, Barton R, Steen N, Campbell M. Is untargeted outreach visiting in primary care effective? A pragmatic randomized controlled trial. J Public Health Med. 2001;23(2):109-13.
45. Hebert C, Beaumont J, Schwartz G, Robicsek A. The influence of context on antimicrobial prescribing for febrile respiratory illness: a cohort study. Ann Intern Med. 2012;157(3):160-9.
46. Hernandez-Santiago V, Marwick CA, Patton A, Davey PG, Donnan PT, Guthrie B. Time series analysis of the impact of an intervention in Tayside, Scotland to reduce primary care broad-spectrum antimicrobial use. J Antimicrob Chemother. 2015;70(8):2397-404.
47. Hickman DE, Stebbins MR, Hanak JR, Guglielmo BJ. Pharmacy-based intervention to reduce antibiotic use for acute bronchitis. Ann Pharmacother. 2003;37(2):187-91.
48. Hui Min Lee M STPD, Huixin Huang J, I-Cheng Chen M, Hui Goh E, Jiang L. Efficacy of a patient-based health education intervention in reducing antibiotic use for acute upper respiratory tract infections in the private sector primary care setting in Singapore. Antimicrob Agents Chemother. 2017;61(5).
49. Ilett KF, Johnson S, Greenhill G, Mullen L, Brockis J, Golledge CL, et al. Modification of general practitioner prescribing of antibiotics by use of a therapeutics adviser (academic detailer). Br J Clin Pharmacol. 2000;49(2):168-73.
50. Ives TJ, Frey JJ, Furr SJ, Bentz EJ. Effect of an educational intervention on oral cephalosporin use in primary care. Arch Intern Med. 1987;147(1):44-7.
51. Jakobsen KA, Melbye H, Kelly MJ, Ceynowa C, Molstad S, Hood K, et al. Influence of CRP testing and clinical findings on antibiotic prescribing in adults presenting with acute cough in primary care. Scand J Prim Health Care. 2010;28(4):229-36.
52. Jones NF, Marshall R. Evaluation of an electronic general-practitioner-based syndromic surveillance system--Auckland, New Zealand, 2000-2001. MMWR Suppl. 2004;53:173-8.
53. Juzych NS, Banerjee M, Essenmacher L, Lerner SA. Improvements in antimicrobial prescribing for treatment of upper respiratory tract infections through provider education. J Gen Intern Med. 2005;20(10):901-5.
54. Kavanagh KE, O'Shea E, Halloran R, Cantillon P, Murphy AW. A pilot study of the use of near-patient C-Reactive Protein testing in the treatment of adult respiratory tract infections in one Irish general practice. BMC Fam Pract. 2011;12:93.
55. Lagerlov P, Loeb M, Andrew M, Hjortdahl P. Improving doctors' prescribing behaviour through reflection on guidelines and prescription feedback: a randomised controlled study. Qual Health Care. 2000;9(3):159-65.
56. Legare F, Labrecque M, Cauchon M, Castel J, Turcotte S, Grimshaw J. Training family physicians in shared decision-making to reduce the overuse of antibiotics in acute respiratory infections: a cluster randomized trial. CMAJ. 2012;184(13):E726-34.
57. Linder JA, Schnipper JL, Tsurikova R, Yu T, Volk LA, Melnikas AJ, et al. Documentation-based clinical decision support to improve antibiotic prescribing for acute respiratory infections in primary care: a cluster randomised controlled trial. Inform Prim Care. 2009;17(4):231-40.
58. Linder JA, Schnipper JL, Tsurikova R, Yu DT, Volk LA, Melnikas AJ, et al. Electronic health record feedback to improve antibiotic prescribing for acute respiratory infections. Am J Manag Care. 2010;16(12 Suppl HIT):e311-9.
59. Little P, Williamson I, Warner G, Gould C, Gantley M, Kinmonth AL. Open randomised trial of prescribing strategies in managing sore throat. BMJ. 1997;314(7082):722-7.
60. Little P, Gould C, Williamson I, Moore M, Warner G, Dunleavey J. Pragmatic randomised controlled trial of two prescribing strategies for childhood acute otitis media. BMJ. 2001;322(7282):336-42.

61. Little P, Rumsby K, Kelly J, Watson L, Moore M, Warner G, et al. Information leaflet and antibiotic prescribing strategies for acute lower respiratory tract infection: a randomized controlled trial. JAMA. 2005;293(24):3029-35.
62. Little P, Stuart B, Francis N, Douglas E, Tonkin-Crine S, Anthierens S, et al. Effects of internet-based training on antibiotic prescribing rates for acute respiratory-tract infections: a multinational, cluster, randomised, factorial, controlled trial. Lancet. 2013;382(9899):1175-82.
63. Llor C, Madurell J, Balague-Corbella M, Gomez M, Cots JM. Impact on antibiotic prescription of rapid antigen detection testing in acute pharyngitis in adults: a randomised clinical trial. Br J Gen Pract. 2011;61(586):e244-51.
64. Llor C, Bjerrum L, Arranz J, Garcia G, Cots JM, Gonzalez Lopez-Valcarcel B, et al. C-reactive protein testing in patients with acute rhinosinusitis leads to a reduction in antibiotic use. Fam Pract. 2012;29(6):653-8.
65. Llor C, Cots JM, Lopez-Valcarcel BG, Arranz J, Garcia G, Ortega J, et al. Interventions to reduce antibiotic prescription for lower respiratory tract infections: Happy Audit study. Eur Respir J. 2012;40(2):436-41.
66. Lundborg CS, Hensjo LO, Gustafsson LL. "Academic drug-detailing": from project to practice in a Swedish urban area. Eur J Clin Pharmacol. 1997;52(3):167-72.
67. Lundborg CS, Wahlstrom R, Oke T, Tomson G, Diwan VK. Influencing prescribing for urinary tract infection and asthma in primary care in Sweden: a randomized controlled trial of an interactive educational intervention. J Clin Epidemiol. 1999;52(8):801-12.
68. Macfarlane JT, Holmes WF, Macfarlane RM. Reducing reconsultations for acute lower respiratory tract illness with an information leaflet: a randomized controlled study of patients in primary care. Br J Gen Pract. 1997;47(424):719-22.
69. Macfarlane J, Holmes W, Gard P, Thornhill D, Macfarlane R, Hubbard R. Reducing antibiotic use for acute bronchitis in primary care: blinded, randomised controlled trial of patient information leaflet. BMJ. 2002;324(7329):91-4.
70. Madridejos-Mora R, Amado-Guirado E, Perez-Rodriguez MT. Effectiveness of the combination of feedback and educational recommendations for improving drug prescription in general practice. Med Care. 2004;42(7):643-8.
71. Magrini N, Formoso G, Capelli O, Maestri E, Nonino F, Paltrinieri B, et al. Long term effectiveness on prescribing of two multifaceted educational interventions: results of two large scale randomized cluster trials. PLoS One. 2014;9(10):e109915.
72. Mainous AG, 3rd, Hueston WJ, Love MM, Evans ME, Finger R. An evaluation of statewide strategies to reduce antibiotic overuse. Fam Med. 2000;32(1):22-9.
73. Margolis CZ, Warshawsky SS, Goldman L, Dagan O, Wirtschafter D, Pliskin JS. Computerized algorithms and pediatricians' management of common problems in a community clinic. Acad Med. 1992;67(4):282-4.
74. Martens JD, Winkens RA, van der Weijden T, de Bruyn D, Severens JL. Does a joint development and dissemination of multidisciplinary guidelines improve prescribing behaviour: a pre/post study with concurrent control group and a randomised trial. BMC Health Serv Res. 2006;6:145.
75. McGinn TG, McCullagh L, Kannry J, Knaus M, Sofianou A, Wisnivesky JP, et al. Efficacy of an evidence-based clinical decision support in primary care practices: a randomized clinical trial. JAMA Intern Med. 2013;173(17):1584-91.
76. Meeker D, Linder JA, Fox CR, Friedberg MW, Persell SD, Goldstein NJ, et al. Effect of Behavioral Interventions on Inappropriate Antibiotic Prescribing Among Primary Care Practices: A Randomized Clinical Trial. JAMA. 2016;315(6):562-70.
77. Melbye H, Aaraas I, Fleten N, Kolstrup N, Mikalsen JI. [The value of C-reactive protein testing in suspected lower respiratory tract infections. A study from general practice on the effect of a rapid test on antibiotic research and course of the disease in adults]. Tidsskr Nor Laegeforen. 1995;115(13):1610-5.

78. Naughton C, Feely J, Bennett K. A RCT evaluating the effectiveness and cost-effectiveness of academic detailing versus postal prescribing feedback in changing GP antibiotic prescribing. J Eval Clin Pract. 2009;15(5):807-12.
79. Ndefo UA, Norman R, Henry A. Academic Detailing Has a Positive Effect on Prescribing and Decreasing Prescription Drug Costs: A Health Plan's Perspective. Am Health Drug Benefits. 2017;10(3):129-33.
80. Olson DR, Paladini M, Lober WB, Buckeridge DL, Group IDW. Applying a New Model for Sharing Population Health Data to National Syndromic Influenza Surveillance: DiSTRIBuTE Project Proof of Concept, 2006 to 2009. PLoS Curr. 2011;3:RRN1251.
81. Peterson GM, Stanton LA, Bergin JK, Chapman GA. Improving the prescribing of antibiotics for urinary tract infection. J Clin Pharm Ther. 1997;22(2):147-53.
82. Price D, Chan D, Greaves N. Physician surveillance of influenza: collaboration between primary care and public health. Can Fam Physician. 2014;60(1):e7-15.
83. Pshetizky Y, Naimer S, Shvartzman P. Acute otitis media--a brief explanation to parents and antibiotic use. Fam Pract. 2003;20(4):417-9.
84. Razon Y, Ashkenazi S, Cohen A, Hering E, Amzel S, Babilsky H, et al. Effect of educational intervention on antibiotic prescription practices for upper respiratory infections in children: a multicentre study. J Antimicrob Chemother. 2005;56(5):937-40.
85. Regev-Yochay G, Raz M, Dagan R, Roizin H, Morag B, Hetman S, et al. Reduction in antibiotic use following a cluster randomized controlled multifaceted intervention: the Israeli judicious antibiotic prescription study. Clin Infect Dis. 2011;53(1):33-41.
86. Roque F, Teixeira-Rodrigues A, Breitenfeld L, Pineiro-Lamas M, Figueiras A, Herdeiro MT. Decreasing antibiotic use through a joint intervention targeting physicians and pharmacists. Future Microbiol. 2016;11:877-86.
87. Saint S, Scholes D, Fihn SD, Farrell RG, Stamm WE. The effectiveness of a clinical practice guideline for the management of presumed uncomplicated urinary tract infection in women. Am J Med. 1999;106(6):636-41.
88. Santoso B, Suryawati S, Prawaitasari JE. Small group intervention vs formal seminar for improving appropriate drug use. Soc Sci Med. 1996;42(8):1163-8.
89. Schaffner W, Ray WA, Federspiel CF, Miller WO. Improving antibiotic prescribing in office practice. A controlled trial of three educational methods. JAMA. 1983;250(13):1728-32.
90. Shah N RJ, Konchak C et al. What's going around? A prospective cluster randomised trial to evaluate a novel, real-time, syndromic surveillance tool's effect on clinical decision making amongst primary care providers. Open Forum Infect Dis (Suppl 1): S78. 2014.
91. Simonsen GS CT, Ilkbrekke L et al. Real-time local epidemiological data through the internet as a management tool for infections in the community [Abstract P1306]. 21st European Congress of Clinical Microbiology and Infectious Diseases; Milan, Italy, May 7-10, 20112011.
92. Smabrekke L, Berild D, Giaever A, Myrbakk T, Fuskevag A, Ericson JU, et al. Educational intervention for parents and healthcare providers leads to reduced antibiotic use in acute otitis media. Scand J Infect Dis. 2002;34(9):657-9.
93. Smeets HM, Kuyvenhoven MM, Akkerman AE, Welschen I, Schouten GP, van Essen GA, et al. Intervention with educational outreach at large scale to reduce antibiotics for respiratory tract infections: a controlled before and after study. Fam Pract. 2009;26(3):183-7.
94. Stewart J, Pilla J, Dunn L. Pilot study for appropriate anti-infective community therapy. Effect of a guideline-based strategy to optimize use of antibiotics. Can Fam Physician. 2000;46:851-9.
95. Sustersic M, Jeannet E, Cozon-Rein L, Marechaux F, Genty C, Foote A, et al. Impact of information leaflets on behavior of patients with gastroenteritis or tonsillitis: a cluster randomized trial in French primary care. J Gen Intern Med. 2013;28(1):25-31.

96. Taylor JA, Kwan-Gett TS, McMahon EM, Jr. Effectiveness of a parental educational intervention in reducing antibiotic use in children: a randomized controlled trial. Pediatr Infect Dis J. 2005;24(6):489-93.
97. Temte JL, Shult PA, Kirk CJ, Amspaugh J. Effects of viral respiratory disease education and surveillance on antibiotic prescribing. Fam Med. 1999;31(2):101-6.
98. van Driel ML, Coenen S, Dirven K, Lobbestael J, Janssens I, Van Royen P, et al. What is the role of quality circles in strategies to optimise antibiotic prescribing? A pragmatic cluster-randomised controlled trial in primary care. Qual Saf Health Care. 2007;16(3):197-202.
99. Vellinga A, Galvin S, Duane S, Callan A, Bennett K, Cormican M, et al. Intervention to improve the quality of antimicrobial prescribing for urinary tract infection: a cluster randomized trial. CMAJ. 2016;188(2):108-15.
100. Veninga CC, Denig P, Zwaagstra R, Haaijer-Ruskamp FM. Improving drug treatment in general practice. J Clin Epidemiol. 2000;53(7):762-72.
101. Vervloet M, Meulepas MA, Cals JW, Eimers M, van der Hoek LS, van Dijk L. Reducing antibiotic prescriptions for respiratory tract infections in family practice: results of a cluster randomized controlled trial evaluating a multifaceted peer-group-based intervention. NPJ Prim Care Respir Med. 2016;26:15083.
102. Vinnard C, Linkin DR, Localio AR, Leonard CE, Teal VL, Fishman NO, et al. Effectiveness of interventions in reducing antibiotic use for upper respiratory infections in ambulatory care practices. Popul Health Manag. 2013;16(1):22-7.
103. Weiss K, Blais R, Fortin A, Lantin S, Gaudet M. Impact of a multipronged education strategy on antibiotic prescribing in Quebec, Canada. Clin Infect Dis. 2011;53(5):433-9.
104. Welschen I, Kuyvenhoven MM, Hoes AW, Verheij TJ. Effectiveness of a multiple intervention to reduce antibiotic prescribing for respiratory tract symptoms in primary care: randomised controlled trial. BMJ. 2004;329(7463):431.
105. Wheeler JG, Fair M, Simpson PM, Rowlands LA, Aitken ME, Jacobs RF. Impact of a waiting room videotape message on parent attitudes toward pediatric antibiotic use. Pediatrics. 2001;108(3):591-6.
106. Wilf-Miron R, Ron N, Ishai S, Chory H, Abboud L, Peled R. Reducing the volume of antibiotic prescriptions: a peer group intervention among physicians serving a community with special ethnic characteristics. J Manag Care Pharm. 2012;18(4):324-8.
107. Wilson EJ, Nasrin D, Dear KB, Douglas RM. Changing GPs' antibiotic prescribing: a randomised controlled trial. Commun Dis Intell Q Rep. 2003;27 Suppl:S32-8.
108. Worrall G, Kettle A, Graham W, Hutchinson J. Postdated versus usual delayed antibiotic prescriptions in primary care: Reduction in antibiotic use for acute respiratory infections? Can Fam Physician. 2010;56(10):1032-6.
109. Zelicoff A, Brillman J, Forslund DW, George JE, Zink S, Koenig S, et al. The Rapid Syndrome Validation Project (RSVP). Proc AMIA Symp. 2001:771-5.
